# The Brown Algal Phlorotannin Dieckol Attenuates Amyloid-β Oligomer-Induced Oxidative DNA Damage and Senescence-like Stress in Human Neuron-like Cells

**DOI:** 10.3390/md24090320

**Published:** 2026-09-11

**Authors:** Yu-Ru Lee, Mei Chou Lai, Yu-Shun Tzeng, I-Min Liu

**Affiliations:** 1 Department of Pet Care and Grooming, College of Pharmacy and Health Care, Tajen University, Pingtung County 90741, Taiwan; 2 Department of Pharmacy and Master Program, College of Pharmacy and Health Care, Tajen University, Pingtung County 90741, Taiwan; 3 Department of Medicine, College of Medicine, National Cheng Kung University, Tainan 70101, Taiwan

**Keywords:** amyloid-β oligomers, senescence-like alterations, dieckol, brown algae, DNA damage response, mitochondrial oxidative stress

## Abstract

Amyloid-β oligomers (AβOs) induce oxidative and genomic stress that may promote neuronal senescence-like alterations in Alzheimer’s disease. Dieckol, a brown algal phlorotannin, has antioxidant and neuroprotective properties, but its effects on AβO-induced senescence-like stress remain unclear. Differentiated SH-SY5Y cells were pretreated with dieckol (10–50 μmol/L) or the receptor for advanced glycation end products (RAGE) antagonist azeliragon (100 μmol/L) for 24 h, followed by AβO (20 μmol/L) exposure for 24 h. Dieckol concentration-dependently preserved cell viability, as indirectly assessed by the MTT assay and reduced mitochondrial superoxide accumulation. Dieckol also decreased senescence-associated β-galactosidase positivity, H3K9me2/3-associated chromatin remodeling, and interleukin-1β, interleukin-6, and tumor necrosis factor-α secretion while partially restoring telomerase activity. This restoration was consistent with improved cellular stress resilience in the differentiated neuron-like model. Furthermore, dieckol reduced 8-hydroxy-2′-deoxyguanosine and γ-H2AX accumulation and attenuated ATM–Chk2–p53 DNA damage response signaling. Azeliragon elicited broadly similar responses; however, these similarities do not establish RAGE dependence or a shared mechanism of action. Collectively, dieckol concurrently attenuated mitochondrial oxidative stress, DNA damage response signaling, and senescence-associated alterations. Although these parallel effects support a possible relationship among these processes, their causal and temporal relationships remain to be established.

## 1. Introduction

Cellular senescence has emerged as an active, maladaptive aging-associated stress program that may perpetuate neurodegenerative injury. It is characterized by coordinated alterations, including increased senescence-associated β-galactosidase (SA-β-gal) activity, persistent DNA damage signaling, chromatin remodeling, activation of growth arrest-associated pathways, reduced telomere-maintenance capacity, and secretion of pro-inflammatory mediators collectively known as the senescence-associated secretory phenotype (SASP) [1]. In the central nervous system, the accumulation of senescence-like alterations may disrupt neuronal homeostasis, amplify inflammatory signaling, and propagate stress responses within the local microenvironment. These processes may contribute to the chronic and self-reinforcing nature of Alzheimer’s disease (AD) neurodegeneration alongside established pathological features, including amyloid plaques, neurofibrillary tangles, synaptic dysfunction, and neuronal loss [2].

Amyloid-β (Aβ) deposition is a pathological hallmark of AD, with the aggregation-prone Aβ_42_ isoform constituting a major component of amyloid plaques [3]. In addition to plaque deposition, soluble Aβ_42_ oligomers (AβOs) are particularly neurotoxic and can induce mitochondrial dysfunction, oxidative stress, inflammation, and genomic damage [4,5]. These effects may promote the accumulation of phosphorylated histone H2AX (γ-H2AX), activation of the ataxia telangiectasia mutated (ATM)–checkpoint kinase 2 (Chk2)–p53 signaling pathway, impaired cellular repair, and increased secretion of SASP-associated factors [6]. Collectively, these interconnected responses suggest that Aβ toxicity extends beyond amyloid deposition to encompass persistent neuronal stress and senescence-associated alterations. One potential mediator of these effects is the receptor for advanced glycation end products (RAGE), a pattern-recognition receptor that binds to several stress-associated ligands, including advanced glycation end products (AGEs) and Aβ [7]. In AD, RAGE may function not merely as a passive Aβ-binding receptor but as a pathological signaling hub that facilitates Aβ transport and accumulation while linking extracellular amyloid stress to intracellular pathways associated with oxidative injury, inflammatory amplification, blood–brain barrier dysfunction, and neuronal damage [8]. Accordingly, pharmacological modulation of the Aβ–RAGE axis may help attenuate receptor-associated injury signaling and the establishment of persistent neuronal stress phenotypes, in addition to influencing Aβ accumulation [8].

Marine algae are a rich source of structurally diverse bioactive compounds with potential applications in neurodegenerative disease research [9]. Brown algae are particularly abundant in phlorotannins, a class of marine polyphenols composed of polymerized phloroglucinol units [10]. Phlorotannins have attracted interest as potential neuroprotective compounds because of their antioxidant and anti-inflammatory activities and their reported effects on multiple processes implicated in neurodegenerative disease [11]. Their relatively high degree of polymerization and ether-linked structures may enhance electron donation and redox stability, thereby contributing to their free-radical-scavenging capacity [11]. Preclinical evidence further suggests that phlorotannins may influence Aβ pathology at several levels, including inhibition of BACE1 activity, suppression of Aβ production, interference with Aβ oligomerization and fibrillization, and attenuation of Aβ-associated neurotoxicity [11].

Dieckol (C_36_H_22_O_18_), a major phlorotannin isolated from edible brown algae such as Ecklonia cava, has attracted considerable attention because of its antioxidant, anti-inflammatory, antiglycation, and neuroprotective properties [12]. Previous studies have shown that dieckol reduces extracellular and intracellular Aβ accumulation in Swedish mutant amyloid precursor protein-overexpressing N2a cells, potentially through the regulation of proteins involved in amyloid precursor protein processing [13]. Dieckol has also been reported to inhibit AGE–RAGE interactions, reduce intracellular reactive oxygen species generation and AGE accumulation, and protect cells against stress-associated injury [14]. Collectively, these findings suggest that dieckol may modulate both Aβ-associated processes and RAGE-related stress responses relevant to AD pathology. However, despite its reported effects on Aβ production and AGE–RAGE interactions, whether dieckol attenuates AβO-induced oxidative, genomic, and senescence-like stress responses in neuronal cells—and whether RAGE-associated signaling may contribute to these effects—remains unclear.

To address this question, we established an in vitro AD-related neuronal injury model using differentiated SH-SY5Y cells exposed directly to AβO. Differentiated SH-SY5Y cells are widely used as a human neuron-like model and provide a reproducible platform for investigating Aβ-associated neuronal stress [15]. Unlike amyloid-producing cell models, direct AβO exposure allows the downstream cellular consequences of extracellular amyloid stress to be examined independently of changes in endogenous Aβ production. This model is therefore suitable for determining whether dieckol protects neuron-like cells against AβO-induced oxidative, genomic, and senescence-like alterations rather than merely modulating Aβ generation. Azeliragon (TTP488 or PF-04494700), an orally bioavailable small-molecule RAGE antagonist that binds to RAGE and inhibits its interactions with multiple ligands, was included as a RAGE-targeting pharmacological comparator [16]. By comparing the response patterns elicited by dieckol and azeliragon, we aimed to determine whether dieckol attenuates AβO-induced neuronal senescence-like alterations and whether its protective effects are consistent with the possible involvement of RAGE-associated oxidative and inflammatory signaling.

## 2. Results

### 2.1. Dieckol Attenuated the AβO-Induced Reduction in Cell Viability in SH-SY5Y Cells

**Figure 1:**
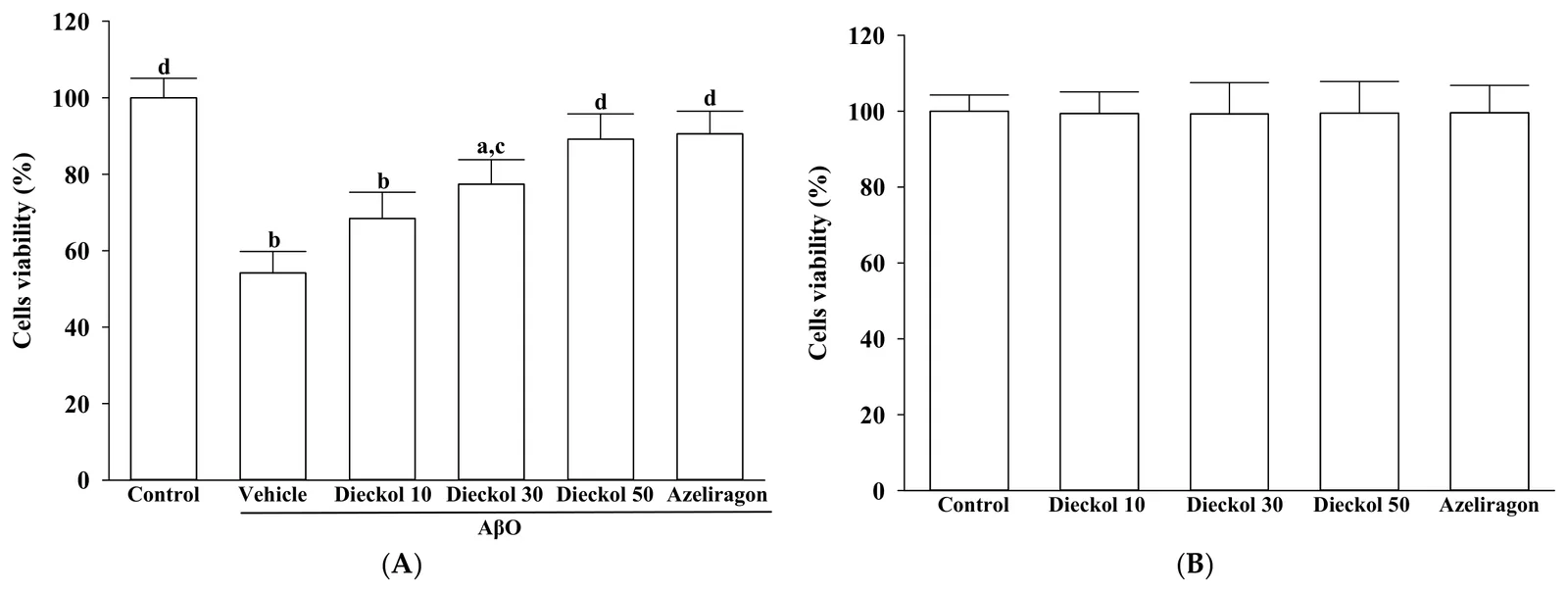
Protective effect of dieckol against the AβO-induced reduction in cell viability in SH-SY5Y cells. Cells were pretreated with dieckol (10, 30, or 50 μmol/L) or azeliragon (100 μmol/L) for 24 h and subsequently exposed to AβO (20 μmol/L) or vehicle for an additional 24 h in the continued presence of the corresponding compound. In the group labels, “Dieckol 10,” “Dieckol 30,” and “Dieckol 50” denote treatment with dieckol at final concentrations of 10, 30, and 50 μmol/L, respectively. Control cells received vehicle alone without AβO, dieckol, or azeliragon. (**A**) Cell viability under AβO exposure conditions. The AβO-exposed vehicle group received AβO without dieckol or azeliragon. (**B**) Effects of dieckol or azeliragon alone on cell viability in the absence of AβO. Cell viability as indirectly assessed by the MTT assay and expressed as a percentage of the vehicle-treated control value. Data are presented as the mean ± SD of five independent biological experiments (*n* = 5). Each experiment was performed in technical triplicate, and the mean of the technical replicates was treated as one biological replicate for statistical analysis. Statistical comparisons were performed using one-way ANOVA followed by Tukey’s multiple-comparisons test. a*p* < 0.05, b*p* < 0.01 vs. vehicle-treated control cells (control); c*p* < 0.05, d*p* < 0.01 vs. the AβO-exposed vehicle group. No significant differences were observed among the groups in panel (**B**).

Exposure to AβO (20 μmol/L) for 24 h reduced relative MTT activity to 53.2% of the vehicle-treated control level. Pretreatment with dieckol attenuated this reduction in a concentration-related manner, increasing MTT-derived cellular metabolic activity to 68.4%, 77.4%, and 89.2% at concentrations of 10, 30, and 50 μmol/L, respectively (Figure 1A). Compared with the AβO (20 μmol/L)-exposed vehicle group, cellular metabolic activity was significantly increased by 30 and 50 μmol/L dieckol, whereas the increase observed with 10 μmol/L dieckol did not reach statistical significance. The greatest protective effect was observed with 50 μmol/L dieckol, which restored cellular metabolic activity to a level similar to that achieved with azeliragon (100 μmol/L; 90.6%). In the absence of AβO (20 μmol/L), neither dieckol nor azeliragon significantly affected cellular metabolic activity, indicating no evident cytotoxicity at the concentrations tested under these experimental conditions (Figure 1B).

### 2.2. Dieckol Attenuated AβO-Induced Mitochondrial Superoxide Accumulation in SH-SY5Y Cells

**Figure 2:**
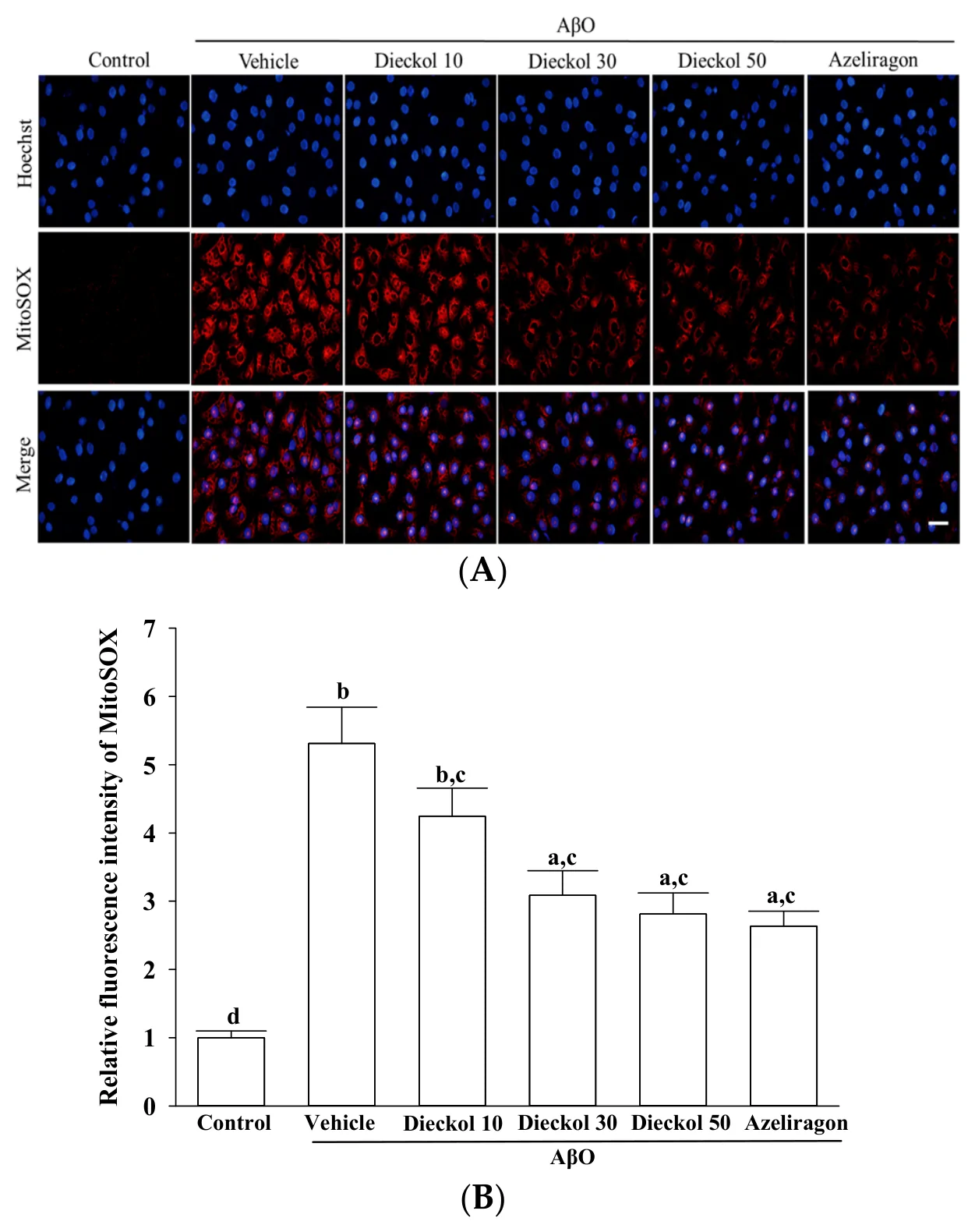
Dieckol attenuated AβO-induced mitochondrial superoxide accumulation in SH-SY5Y cells. Cells were pretreated with dieckol (10, 30, or 50 μmol/L) or azeliragon (100 μmol/L) for 24 h and subsequently exposed to AβO (20 μmol/L) for an additional 24 h in the continued presence of the corresponding compound. In the group labels, “Dieckol 10,” “Dieckol 30,” and “Dieckol 50” denote treatment with dieckol at final concentrations of 10, 30, and 50 μmol/L, respectively. Control cells received vehicle alone without AβO, dieckol, or azeliragon. The AβO-exposed vehicle group received AβO and vehicle without dieckol or azeliragon. (**A**) Representative fluorescence images showing Hoechst-stained nuclei (blue), MitoSOX fluorescence (red), and the corresponding merged images. Scale bar: 50 μm. (**B**) Quantification of relative MitoSOX fluorescence intensity, expressed as fold change relative to the vehicle-treated control group. Data are presented as the mean ± SD of five independent biological experiments (*n* = 5). For each biological experiment, fluorescence intensity was quantified in five randomly selected fields, and the mean value of these fields was treated as one biological replicate for statistical analysis. Statistical comparisons were performed using one-way ANOVA followed by Tukey’s multiple-comparisons test. a*p* < 0.05, b*p* < 0.01 vs. vehicle-treated control cells (control); c*p* < 0.05, d*p* < 0.01 vs. the AβO-exposed vehicle group.

Exposure of differentiated SH-SY5Y cells to AβO (20 μmol/L) markedly increased MitoSOX fluorescence, whereas pretreatment with dieckol attenuated this increase in a concentration-related manner (Figure 2A). Quantitative analysis showed that AβO (20 μmol/L) increased the relative MitoSOX fluorescence intensity to approximately 5.3-fold the untreated control level (Figure 2B). Compared with the AβO (20 μmol/L)-exposed vehicle group, pretreatment with dieckol at 10, 30, and 50 μmol/L significantly reduced the relative fluorescence intensity to approximately 4.2-, 3.1-, and 2.8-fold the untreated control level, respectively. Pretreatment with azeliragon (100 μmol/L) also significantly reduced the relative MitoSOX fluorescence intensity to approximately 2.6-fold the untreated control level.

### 2.3. Dieckol Attenuated AβO-Induced Senescence-like Alterations in SH-SY5Y Cells

**Figure 3:**
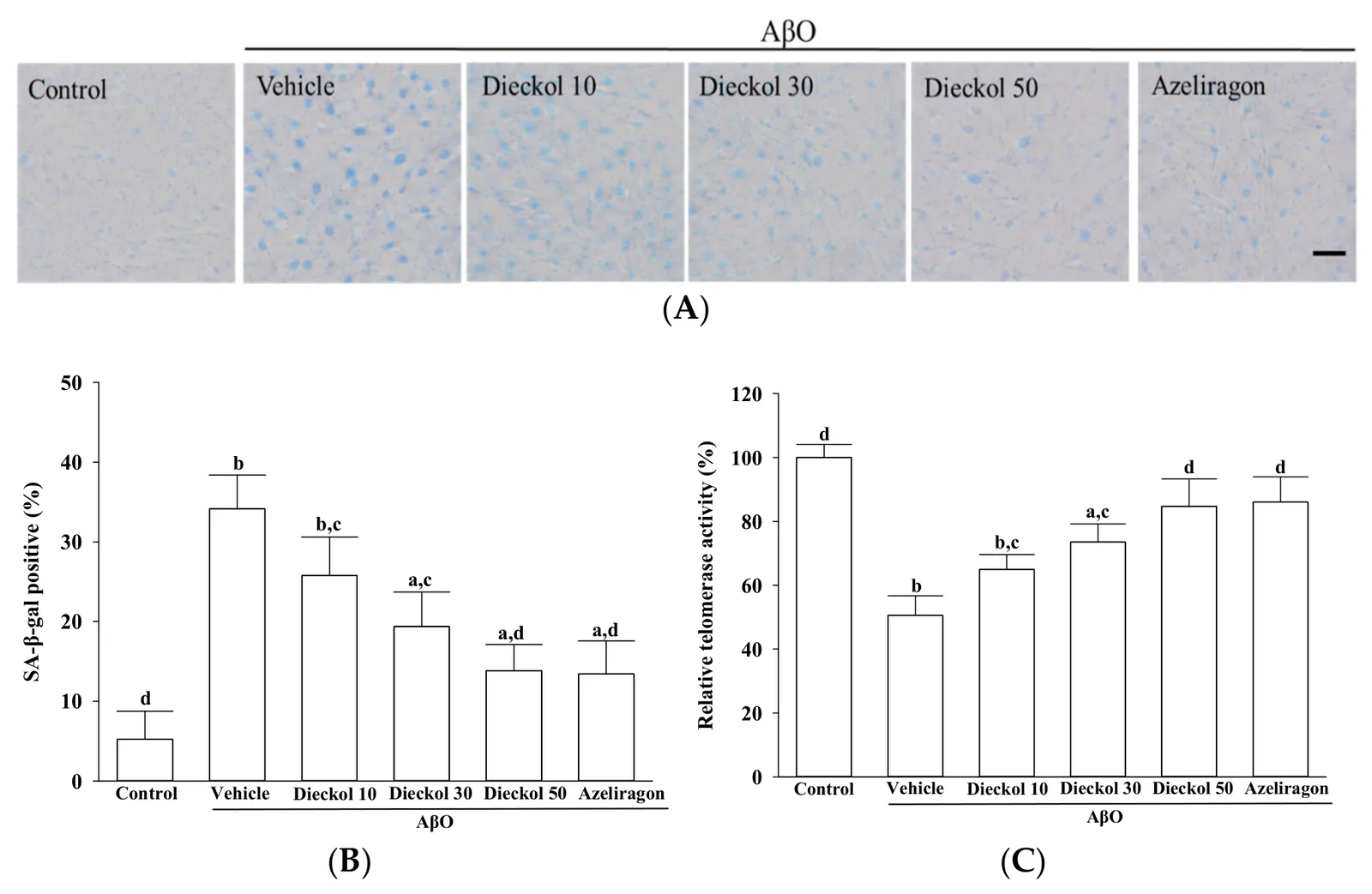
Dieckol attenuated AβO-induced senescence-associated alterations in SH-SY5Y cells. Cells were pretreated with dieckol (10, 30, or 50 μmol/L) or azeliragon (100 μmol/L) for 24 h and subsequently exposed to AβO (20 μmol/L) for an additional 24 h in the continued presence of the corresponding compound. In the group labels, “Dieckol 10,” “Dieckol 30,” and “Dieckol 50” denote treatment with dieckol at final concentrations of 10, 30, and 50 μmol/L, respectively. Control cells received vehicle alone without AβO, dieckol, or azeliragon. The AβO-exposed vehicle group received AβO and vehicle without dieckol or azeliragon. (**A**) Representative images of senescence-associated β-galactosidase (SA-β-gal) staining under the indicated treatment conditions. Cells exhibiting blue cytoplasmic staining were considered SA-β-gal positive. Scale bar: 50 μm. (**B**) Percentage of SA-β-gal-positive cells among the total cells counted. (**C**) Relative telomerase activity, expressed as a percentage of the vehicle-treated control level. Data are presented as the mean ± SD of five independent biological experiments (*n* = 5). For SA-β-gal analysis, SA-β-gal-positive and total cells were counted in five randomly selected fields per biological experiment, and the mean percentage across these fields was treated as one biological replicate for statistical analysis. Telomerase activity was measured in technical triplicate, and the mean of the technical replicates was used as the value for each biological experiment. Statistical comparisons for panels (**B**,**C**) were performed separately using one-way ANOVA followed by Tukey’s multiple-comparisons test. a*p* < 0.05, b*p* < 0.01 vs. vehicle-treated control group (control); c*p* < 0.05, d*p* < 0.01 vs. AβO-exposed vehicle group.

Exposure of differentiated SH-SY5Y cells to AβO (20 μmol/L) markedly increased the proportion of SA-β-gal-positive cells, whereas pretreatment with dieckol attenuated this increase in a concentration-related manner (Figure 3A). Quantitative analysis showed that AβO (20 μmol/L) increased the percentage of SA-β-gal-positive cells from approximately 5% in the vehicle-treated control group to 34% in the AβO (20 μmol/L)-exposed vehicle group (Figure 3B). Compared with the AβO (20 μmol/L)-exposed vehicle group, pretreatment with dieckol at 10, 30, and 50 μmol/L significantly reduced the percentage of SA-β-gal-positive cells to approximately 26%, 19%, and 14%, respectively. Pretreatment with azeliragon (100 μmol/L) also significantly reduced the percentage of SA-β-gal-positive cells to approximately 13%.

AβO (20 μmol/L) exposure significantly decreased telomerase activity to approximately 50% of the vehicle-treated control level (Figure 3C). Pretreatment with dieckol partially restored telomerase activity in a concentration-related manner, increasing it to approximately 65%, 74%, and 85% of the control level at concentrations of 10, 30, and 50 μmol/L, respectively. Pretreatment with azeliragon also significantly increased telomerase activity to approximately 86% of the control level.

### 2.4. Dieckol Attenuated AβO-Induced H3K9me2/3-Associated Chromatin Remodeling and SASP-Associated Cytokine Secretion in SH-SY5Y Cells

**Figure 4:**
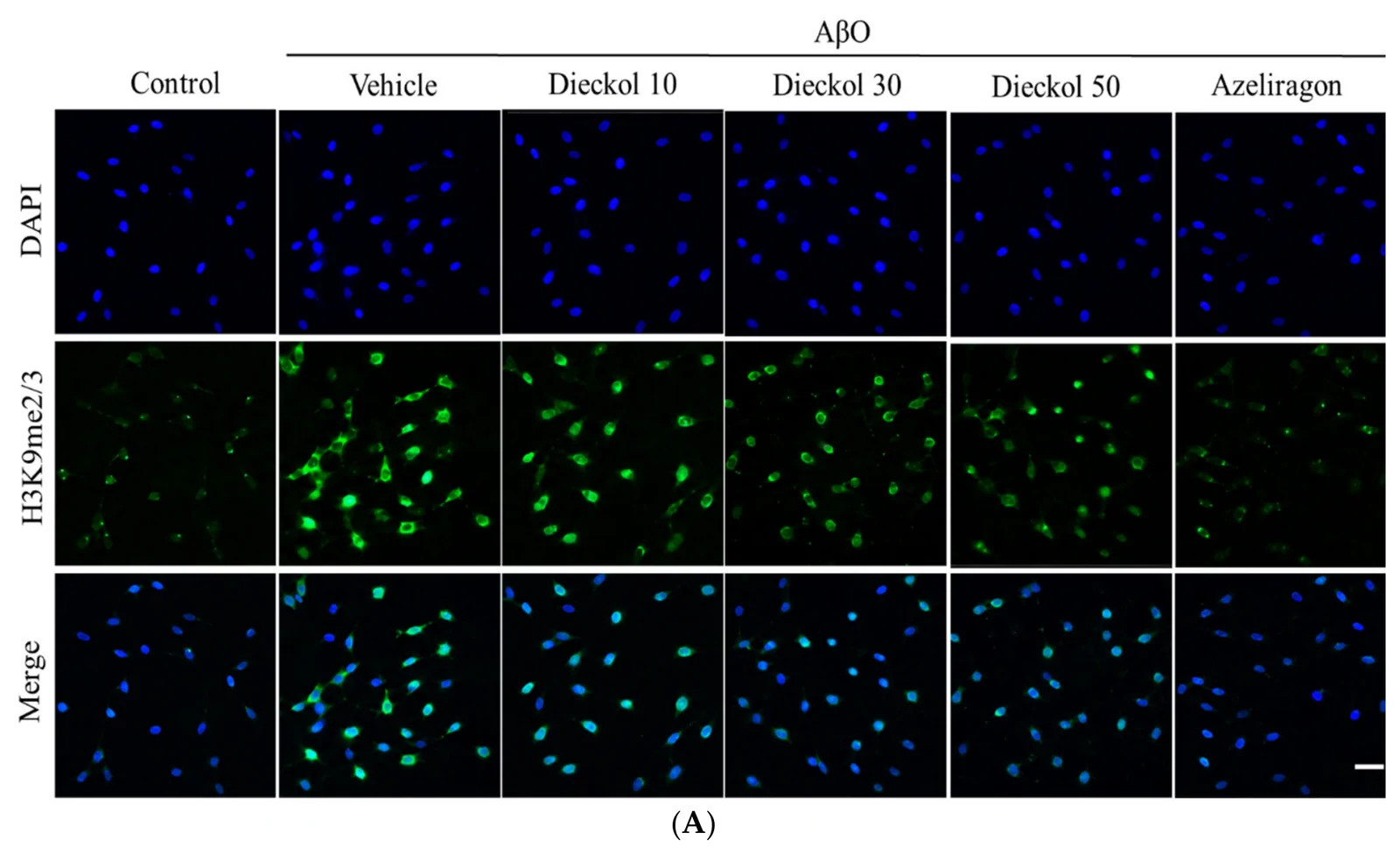

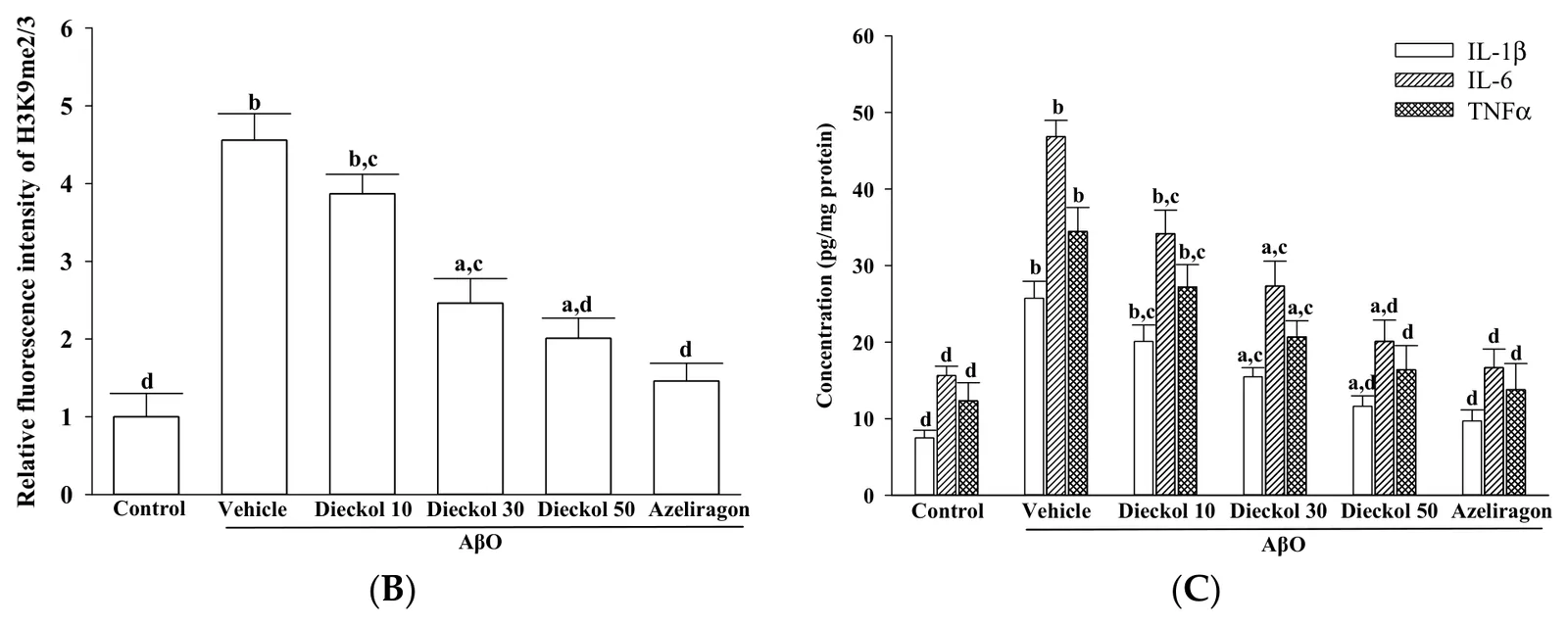
Dieckol attenuated AβO-induced H3K9me2/3-associated chromatin remodeling and SASP-associated cytokine secretion in SH-SY5Y cells. Cells were pretreated with dieckol (10, 30, or 50 μmol/L) or azeliragon (100 μmol/L) for 24 h and subsequently exposed to AβO (20 μmol/L) for an additional 24 h in the continued presence of the corresponding compound. In the group labels, “Dieckol 10,” “Dieckol 30,” and “Dieckol 50” denote treatment with dieckol at final concentrations of 10, 30, and 50 μmol/L, respectively. Control cells received vehicle alone without AβO, dieckol, or azeliragon. The AβO-exposed vehicle group received AβO and vehicle without dieckol or azeliragon. (**A**) Representative immunofluorescence images of H3K9me2/3. Nuclei were counterstained with DAPI (blue), and H3K9me2/3 immunoreactivity is shown in green. Scale bar: 50 μm. (**B**) Quantification of relative H3K9me2/3 fluorescence intensity, expressed as fold change relative to the vehicle-treated control group. (**C**) Concentrations of the pro-inflammatory cytokines IL-1β, IL-6, and TNF-α in the culture supernatants, normalized to total cellular protein and expressed as pg/mg protein. Data are presented as the mean ± SD of five independent biological experiments (*n* = 5). For H3K9me2/3 analysis, fluorescence intensity was quantified in five randomly selected fields per biological experiment, and the mean value of these fields was treated as one biological replicate for statistical analysis. Cytokine concentrations were measured in technical triplicate, and the mean of the technical replicates was used as the value for each biological experiment. Statistical comparisons for panel (**B**) and for each cytokine in panel (**C**) were performed separately using one-way ANOVA followed by Tukey’s multiple-comparisons test. a*p* < 0.05, b*p* < 0.01 vs. vehicle-treated control group (control); c*p* < 0.05, d*p* < 0.01 vs. AβO-exposed vehicle group.

As shown in Figure 4A, exposure of differentiated SH-SY5Y cells to AβO (20 μmol/L) markedly increased nuclear H3K9me2/3 immunofluorescence. Quantitative analysis showed that AβO (20 μmol/L) increased the relative H3K9me2/3 fluorescence intensity to approximately 4.6-fold the vehicle-treated control level (Figure 4B). Compared with the AβO (20 μmol/L)-exposed vehicle group, pretreatment with dieckol at 10, 30, and 50 μmol/L significantly reduced the relative fluorescence intensity to approximately 3.9-, 2.5-, and 2.0-fold the control level, respectively. Pretreatment with azeliragon (100 μmol/L) also significantly reduced the relative H3K9me2/3 fluorescence intensity to approximately 1.5-fold the control level.

AβO (20 μmol/L) exposure also significantly increased the concentrations of the SASP-associated pro-inflammatory cytokines IL-1β, IL-6, and TNF-α in the culture supernatant to approximately 3.3-, 2.9-, and 2.7-fold their respective vehicle-treated control levels (Figure 4C). Pretreatment with dieckol significantly attenuated these increases in a concentration-related manner. At 50 μmol/L, dieckol reduced the concentrations of IL-1β, IL-6, and TNF-α to approximately 1.5-, 1.4-, and 1.5-fold their respective control levels. Pretreatment with azeliragon also significantly reduced the corresponding cytokine concentrations to approximately 1.3-, 1.1-, and 1.1-fold their respective control levels.

### 2.5. Dieckol Attenuated AβO-Induced Oxidative DNA Damage in SH-SY5Y Cells

**Figure 5:**
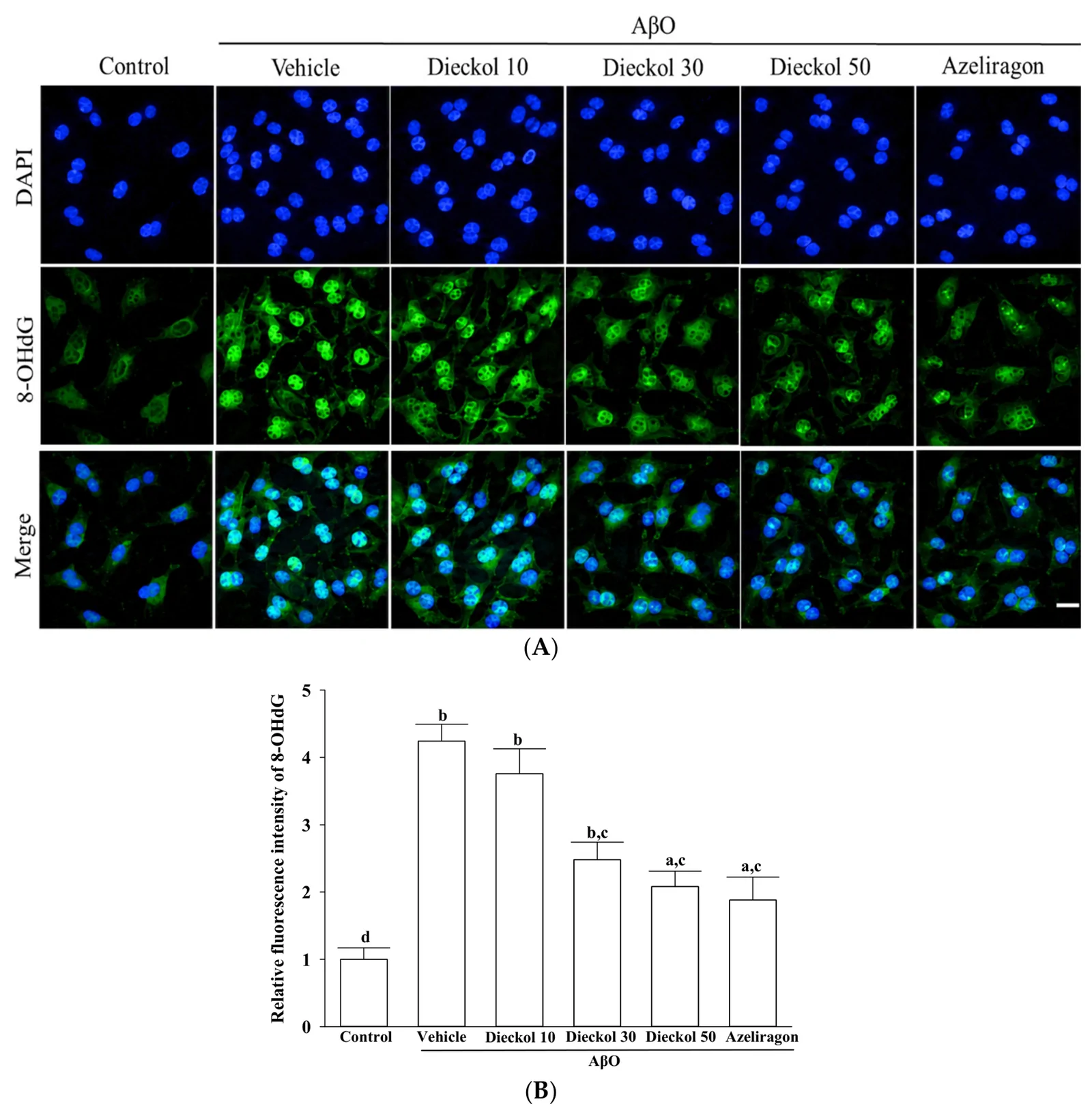
Dieckol attenuated AβO-induced oxidative DNA damage in SH-SY5Y cells. Cells were pretreated with dieckol (10, 30, or 50 μmol/L) or azeliragon (100 μmol/L) for 24 h and subsequently exposed to AβO (20 μmol/L) for an additional 24 h. In the group labels, “Dieckol 10,” “Dieckol 30,” and “Dieckol 50” denote treatment with dieckol at final concentrations of 10, 30, and 50 μmol/L, respectively. Control cells received vehicle alone without AβO, dieckol, or azeliragon. The AβO-exposed vehicle group received AβO and vehicle without dieckol or azeliragon. (**A**) Representative immunofluorescence images of 8-hydroxy-2′-deoxyguanosine (8-OHdG; green). Nuclei were counterstained with DAPI (blue). Scale bar: 10 μm. (**B**) Quantification of the relative 8-OHdG fluorescence intensity, expressed as fold changes relative to the untreated control group. Data are presented as the mean ± SD of five independent biological experiments (*n* = 5). For 8-OHdG analysis, fluorescence intensity was quantified in five randomly selected fields per biological experiment. Statistical comparisons were performed using one-way ANOVA followed by Tukey’s multiple-comparisons test. a*p* < 0.05, b*p* < 0.01 vs. vehicle-treated control group (control); c*p* < 0.05, d*p* < 0.01 vs. AβO-exposed vehicle group.

As shown in Figure 5A, exposure of SH-SY5Y cells to AβO (20 μmol/L) markedly increased 8-OHdG immunofluorescence, whereas pretreatment with dieckol attenuated this increase in a concentration-related manner. Quantitative analysis showed that AβO (20 μmol/L) increased the relative 8-OHdG fluorescence intensity to approximately 4.3-fold relative to the vehicle-treated control value (Figure 5B). Pretreatment with dieckol at 10, 30, and 50 μmol/L reduced the relative fluorescence intensity to approximately 3.8-, 2.5-, and 2.1-fold relative to the control value, respectively. Compared with the AβO (20 μmol/L)-exposed vehicle group, the reductions induced by 30 and 50 μmol/L dieckol were statistically significant, whereas the reduction induced by 10 μmol/L dieckol did not reach statistical significance. Pretreatment with azeliragon (100 μmol/L) also significantly reduced the relative 8-OHdG fluorescence intensity to approximately 1.9-fold relative to the control value.

### 2.6. Dieckol Attenuated AβO-Induced Activation of the DNA Damage Response in SH-SY5Y Cells

**Figure 6:**
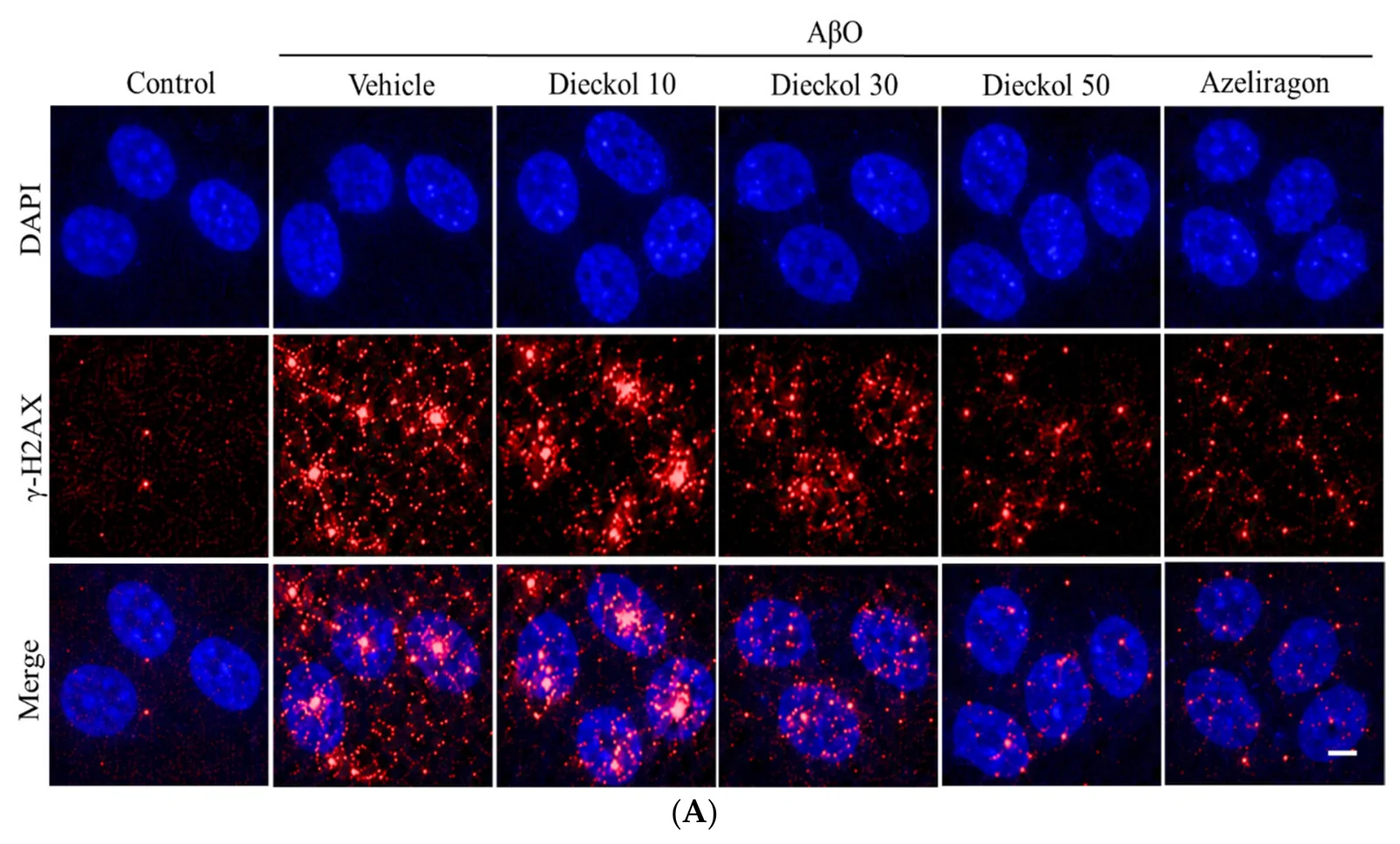

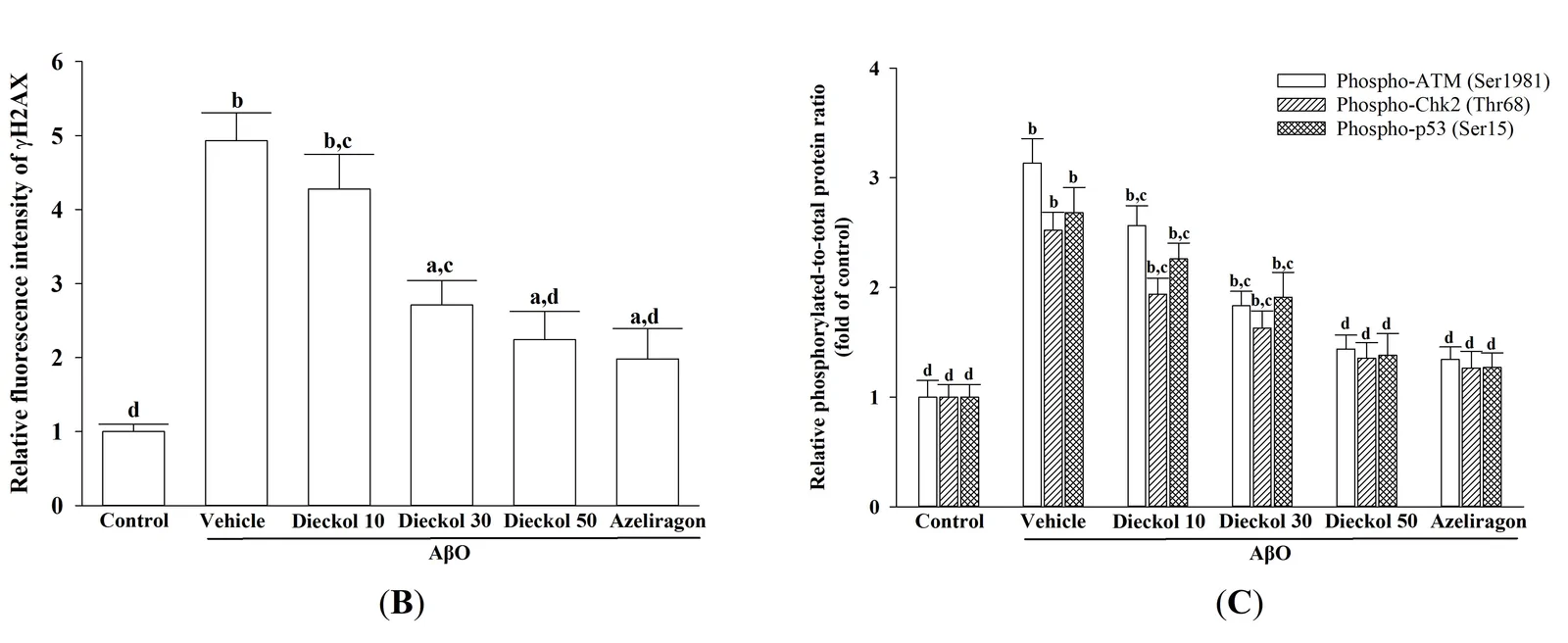
Dieckol attenuated AβO-induced γ-H2AX accumulation and activation of ATM–Chk2–p53 signaling in SH-SY5Y cells. Cells were pretreated with dieckol (10, 30, or 50 μmol/L) or azeliragon (100 μmol/L) for 24 h and subsequently exposed to AβO (20 μmol/L) for an additional 24 h. In the group labels, “Dieckol 10,” “Dieckol 30,” and “Dieckol 50” denote treatment with dieckol at final concentrations of 10, 30, and 50 μmol/L, respectively. Control cells received vehicle alone without AβO, dieckol, or azeliragon. The AβO-exposed vehicle group received AβO and vehicle without dieckol or azeliragon. (**A**) Representative immunofluorescence images of Ser139-phosphorylated histone H2AX (γ-H2AX; red). Nuclei were counterstained with DAPI (blue). Scale bar: 10 μm. (**B**) Quantification of fluorescence within DAPI-defined nuclear regions. Following background subtraction, the integrated fluorescence intensities within these regions were summed for each field and normalized to the number of DAPI-positive nuclei in that field. Values are expressed as fold change relative to the vehicle-treated control group. (**C**) Phospho-ATM (Ser1981)/total ATM, phospho-Chk2 (Thr68)/total Chk2, and phospho-p53 (Ser15)/total p53 ratios, as determined by ELISA and expressed as fold change relative to their respective vehicle-treated control values. Data are presented as the mean ± SD of five independent biological experiments (*n* = 5). For panel (**B**), five randomly selected fields were analyzed per biological experiment, and the normalized field-level values were averaged to generate one value for each biological replicate. For panel (**C**), each biological experiment was measured in technical triplicate, and the technical replicates were averaged before statistical analysis. Statistical comparisons for panel (**B**) and for each phospho-to-total protein ratio in panel (**C**) were performed separately using one-way ANOVA followed by Tukey’s multiple-comparisons test. a*p* < 0.05, b*p* < 0.01 vs. vehicle-treated control group (control); c*p* < 0.05, d*p* < 0.01 vs. AβO-exposed vehicle group.

As shown in Figure 6A, exposure of differentiated SH-SY5Y cells to AβO (20 μmol/L) markedly increased γ-H2AX-associated fluorescence, whereas pretreatment with dieckol attenuated this increase in a concentration-related manner. Quantitative analysis showed that AβO (20 μmol/L) increased the relative γ-H2AX fluorescence intensity to approximately 4.9-fold the vehicle-treated control level (Figure 6B). Compared with the AβO-exposed vehicle group, pretreatment with dieckol at 10, 30, and 50 μmol/L significantly reduced the relative fluorescence intensity to approximately 4.3-, 2.7-, and 2.3-fold the control level, respectively. Pretreatment with azeliragon (100 μmol/L) also significantly reduced the relative γ-H2AX fluorescence intensity to approximately 2.0-fold the control level. Although quantification was restricted to DAPI-defined nuclear regions, appreciable extranuclear γ-H2AX-associated fluorescence was present in the representative images, and discrete intranuclear foci were not consistently resolved. Because no orthogonal assay was performed to validate this staining pattern, these findings should be interpreted as treatment-associated differences in nuclear-region γ-H2AX immunoreactivity rather than as definitive evidence or direct quantification of DNA double-strand breaks.

Consistent with these findings, AβO (20 μmol/L) exposure increased the phospho-ATM (Ser1981)/total ATM, phospho-Chk2 (Thr68)/total Chk2, and phospho-p53 (Ser15)/total p53 ratios to approximately 3.1-, 2.7-, and 2.5-fold their respective vehicle-treated control levels (Figure 6C). Pretreatment with dieckol significantly attenuated these increases in a concentration-related manner. At 10, 30, and 50 μmol/L, dieckol reduced the phospho-ATM/total ATM ratio to approximately 2.6-, 1.8-, and 1.4-fold the control level, respectively; the phospho-Chk2/total Chk2 ratio to approximately 2.3-, 1.9-, and 1.4-fold the control level, respectively; and the phospho-p53/total p53 ratio to approximately 2.0-, 1.6-, and 1.3-fold the control level, respectively. Pretreatment with azeliragon (100 μmol/L) also significantly reduced the phospho-ATM/total ATM, phospho-Chk2/total Chk2, and phospho-p53/total p53 ratios to approximately 1.3-, 1.3-, and 1.2-fold the control level, respectively.

## 3. Discussion

Whereas previous studies demonstrated that dieckol reduces Aβ generation in SweAPP-overexpressing N2a cells and inhibits AGE–RAGE interactions in renal cells [13,14], the present study extends its neuroprotective relevance by demonstrating that dieckol protects differentiated human neuron-like cells against the downstream consequences of direct extracellular AβO exposure. Previous evidence indicates that phlorotannins, including dieckol, can influence multiple stages of Aβ pathology by inhibiting BACE1 activity, interfering with Aβ aggregation, and reducing Aβ-associated neurotoxicity [11]. Dieckol and related phlorotannins have also been reported to suppress ROS accumulation and preserve viability in neuronal cell models exposed to Aβ or other oxidative insults [11]. The present findings identify a coordinated pattern involving reductions in mitochondrial superoxide accumulation, oxidative DNA damage, γ-H2AX accumulation, ATM–Chk2–p53 activation, H3K9me2/3-associated chromatin remodeling, and SASP-associated cytokine secretion, together with partial restoration of telomerase activity. These parallel changes are consistent with a possible relationship among mitochondrial oxidative stress, DNA damage response signaling, and senescence-like stress as a possible interpretative framework for dieckol-mediated protection [17]. However, the present experimental design does not establish the temporal order, directionality, or causal dependence of these events.

Within this interpretative framework, modulation of the ATM–Chk2–p53 pathway may represent a possible link between the antioxidant effects of dieckol and the observed attenuation of senescence-associated alterations. ATM is activated in response to DNA double-strand breaks and oxidative stress and subsequently phosphorylates Chk2 and p53 to coordinate DNA repair, cell-cycle arrest, senescence, or apoptosis, depending on the severity and persistence of cellular injury [18,19]. Accordingly, the parallel reductions in mitochondrial superoxide, γ-H2AX accumulation, 8-OHdG formation, and ATM, Chk2, and p53 phosphorylation following dieckol treatment are consistent with an attenuation of sustained oxidative and genotoxic stress. In this context, γ-H2AX reflects DNA damage response signaling associated with DNA strand breaks, whereas 8-OHdG indicates oxidative modification of DNA [20,21]. These findings should not be interpreted as evidence that dieckol directly inhibits ATM, because suppression of DNA damage surveillance in the presence of unresolved lesions could compromise genomic integrity. Rather, these findings are more consistent with the interpretation that dieckol-mediated attenuation of oxidative and genotoxic stress is accompanied by reduced activation of the ATM–Chk2–p53 cascade; however, the causal directionality of this relationship remains to be established. Although overt chromatin condensation or nuclear fragmentation was not apparent in the representative Hoechst staining, it was used as a nuclear counterstain for nuclear enumeration and normalization of MitoSOX fluorescence rather than as a quantitative assessment of nuclear morphology or apoptosis. Moreover, the MTT assay reflects population-level metabolic activity and provides only an indirect estimate of cell viability, whereas 8-OHdG and γ-H2AX indicate oxidative DNA modification and DNA damage response-associated immunoreactivity, respectively. These molecular alterations can occur without overt chromatin condensation or nuclear fragmentation and do not necessarily indicate progression to apoptosis. Accordingly, the present findings support AβO-induced oxidative and genotoxic stress but do not establish morphological evidence of apoptotic nuclear injury.

Consistent with the attenuation of DNA damage signaling, dieckol modulated lysosomal and chromatin features associated with the AβO-induced senescence-like stress response. Importantly, the 24 h exposure to AβO represents an acute cellular stress paradigm, and no single marker is entirely specific for neuronal senescence, particularly in differentiated neuron-like cells. These findings therefore require interpretation within a multiparametric framework [22]. AβO increased SA-β-gal activity and H3K9me2/3-associated fluorescence, indicating concurrent lysosomal and chromatin alterations [23,24], whereas dieckol attenuated both changes. Although the reduction in H3K9me2/3-associated fluorescence suggests modulation of AβO-induced heterochromatin remodeling, H3K9me2/3 intensity alone does not establish the presence of discrete senescence-associated heterochromatin foci (SAHF). This finding is therefore more appropriately interpreted as evidence of senescence-associated chromatin remodeling. Taken together, the coordinated effects on SA-β-gal activity and H3K9me2/3-associated fluorescence suggest that dieckol attenuates distinct intracellular features of an acute AβO-induced senescence-like stress response; however, they do not provide definitive evidence of progression to a stable or persistent state of cellular or neuronal senescence.

Beyond these lysosomal and chromatin alterations, the effects of dieckol on telomerase activity and inflammatory cytokine secretion further characterize the functional and secretory dimensions of the AβO-induced senescence-like stress response. Although telomere shortening is a classical driver of replicative senescence, the relative telomerase activity measured in the present study should not be regarded as a surrogate for telomere length or integrity. Neither telomere length nor telomere-associated DNA damage was directly assessed; therefore, the reduction in telomerase activity following short-term AβO exposure cannot be interpreted as evidence of rapid telomere erosion or dysfunction, and its restoration by dieckol cannot be considered evidence of telomere elongation or preservation. In addition to its canonical role in telomere maintenance, telomerase may exert noncanonical stress-responsive functions associated with mitochondrial homeostasis, resistance to oxidative injury, and cell survival [25,26]. Accordingly, the observed changes in telomerase activity may reflect modulation of cellular stress-resistance mechanisms rather than direct alterations in telomere dynamics. The restoration of telomerase activity by dieckol is therefore interpreted as being consistent with improved cellular stress resilience, although the underlying telomere-dependent and noncanonical mechanisms were not distinguished in the present study. Direct measurements of telomere length and telomere-associated damage would be required to determine whether telomere dynamics contribute to these effects.

In parallel, AβO increased, whereas dieckol suppressed, the secretion of the selected SASP-associated pro-inflammatory cytokines IL-1β, IL-6, and TNF-α. These changes indicate modulation of inflammatory secretory features accompanying the acute AβO-induced senescence-like stress response. However, the SASP is a heterogeneous and context-dependent secretory program comprising a broader range of cytokines, chemokines, growth factors, and proteases [27,28]. Because only three inflammatory mediators were quantified and no comprehensive cytokine panel or secretome analysis was performed, the present findings do not demonstrate the establishment or global modulation of a complete SASP. Nevertheless, because IL-1β, IL-6, and TNF-α can promote autocrine and paracrine inflammatory signaling, their reduction may limit the reinforcement and propagation of senescence-associated stress within the neural microenvironment. Collectively, the coordinated modulation of lysosomal activity, chromatin organization, telomerase activity, and selected inflammatory mediators supports the interpretation that dieckol attenuates multiple features of the AβO-induced senescence-like stress response while not establishing an effect on telomere dynamics or a complete SASP program.

The broadly similar response patterns produced by dieckol and azeliragon raise the possibility that RAGE-associated signaling contributes to their protective effects; however, their substantial structural, pharmacological, and translational differences warrant careful consideration. Dieckol is a naturally occurring, high-molecular-weight phlorotannin composed of multiple phloroglucinol units and hydroxyl groups. Its high degree of polymerization and ether-linked dibenzodioxin structure have been associated with efficient electron donation, redox stability, and broad antioxidant activity [11]. These structural properties may contribute to its ability to modulate multiple oxidative and stress-associated pathways rather than a single molecular target. In contrast, azeliragon is a synthetic, orally bioavailable small-molecule RAGE antagonist designed to inhibit interactions between RAGE and its ligands, thereby serving as a more target-directed pharmacological comparator [16]. Although azeliragon advanced to phase III clinical evaluation in AD, it failed to demonstrate significant benefits on the primary cognitive and functional endpoints and has not been approved for the treatment of AD [29]. The dieckol concentration range used in the present study (10–50 μmol/L) was selected based on previous cell-based studies to evaluate concentration-dependent responses under controlled in vitro conditions. Its translational relevance remains uncertain because comparable unbound concentrations in plasma or brain tissue following oral or systemic administration have not been established. Although fluorescently labeled dieckol has been detected in the rat cortex and hippocampus following intravenous administration [11], this observation provides only preliminary evidence of central nervous system access and does not define the oral absorption, blood–brain barrier penetration, pharmacokinetics, or therapeutically relevant human brain exposure of unmodified purified dieckol. Likewise, the favorable tolerability reported for standardized *Ecklonia cava* phlorotannin preparations [30] cannot be directly extrapolated to the isolated compound. Thus, the present findings demonstrate cellular activity under experimental conditions but do not establish clinically achievable exposure or therapeutic feasibility. Further investigation is required to characterize the molecular targets, pharmacokinetics, long-term safety, and clinical potential of dieckol. Moreover, the phenotypic similarity between dieckol and azeliragon is consistent with the possible involvement of RAGE-associated signaling but does not demonstrate direct RAGE antagonism or therapeutic equivalence.

Several limitations should be considered when interpreting the present findings. First, the study was conducted exclusively in retinoic acid-differentiated SH-SY5Y cells exposed to AβO under an acute in vitro paradigm. Although this model provides a reproducible platform for investigating Aβ-associated neuronal stress, it does not fully recapitulate the phenotypic complexity of mature human neurons or the multicellular interactions among neurons, astrocytes, microglia, and vascular cells in the AD brain. The findings should therefore be interpreted as evidence of a senescence-like stress response in differentiated neuron-like cells rather than definitive evidence of neuronal senescence. Second, although AβO was prepared using an established oligomerization protocol under standardized conditions, its oligomeric composition and size distribution were not directly characterized, and batch-to-batch consistency was not independently confirmed using biochemical or biophysical methods. Standardized aliquoting, reconstitution, incubation, and immediate use were employed to minimize preparation-to-preparation variability. The findings should therefore be interpreted as responses to an AβO preparation generated under oligomer-promoting conditions using a standardized protocol rather than to a biophysically defined oligomer species. Third, dieckol was administered before AβO exposure; consequently, the present results primarily demonstrate protection against the establishment of AβO-induced injury and do not establish whether dieckol can reverse a pre-existing senescence-like stress response. Fourth, the involvement of RAGE remains mechanistically plausible but inferential. Although dieckol produced a response pattern broadly similar to that of azeliragon, RAGE expression, Aβ–RAGE binding, and receptor-dependent signaling were not directly assessed, and the present findings do not establish direct RAGE antagonism or demonstrate that RAGE is required for dieckol-mediated protection. Nevertheless, within these experimental boundaries, the present findings identify attenuation of the coordinated attenuation of mitochondrial ROS, DNA damage response signaling, and senescence-like alterations as a plausible basis for the neuroprotective effects of dieckol and provide a rationale for further investigation in more physiologically representative models of AD.

**Figure 7:**
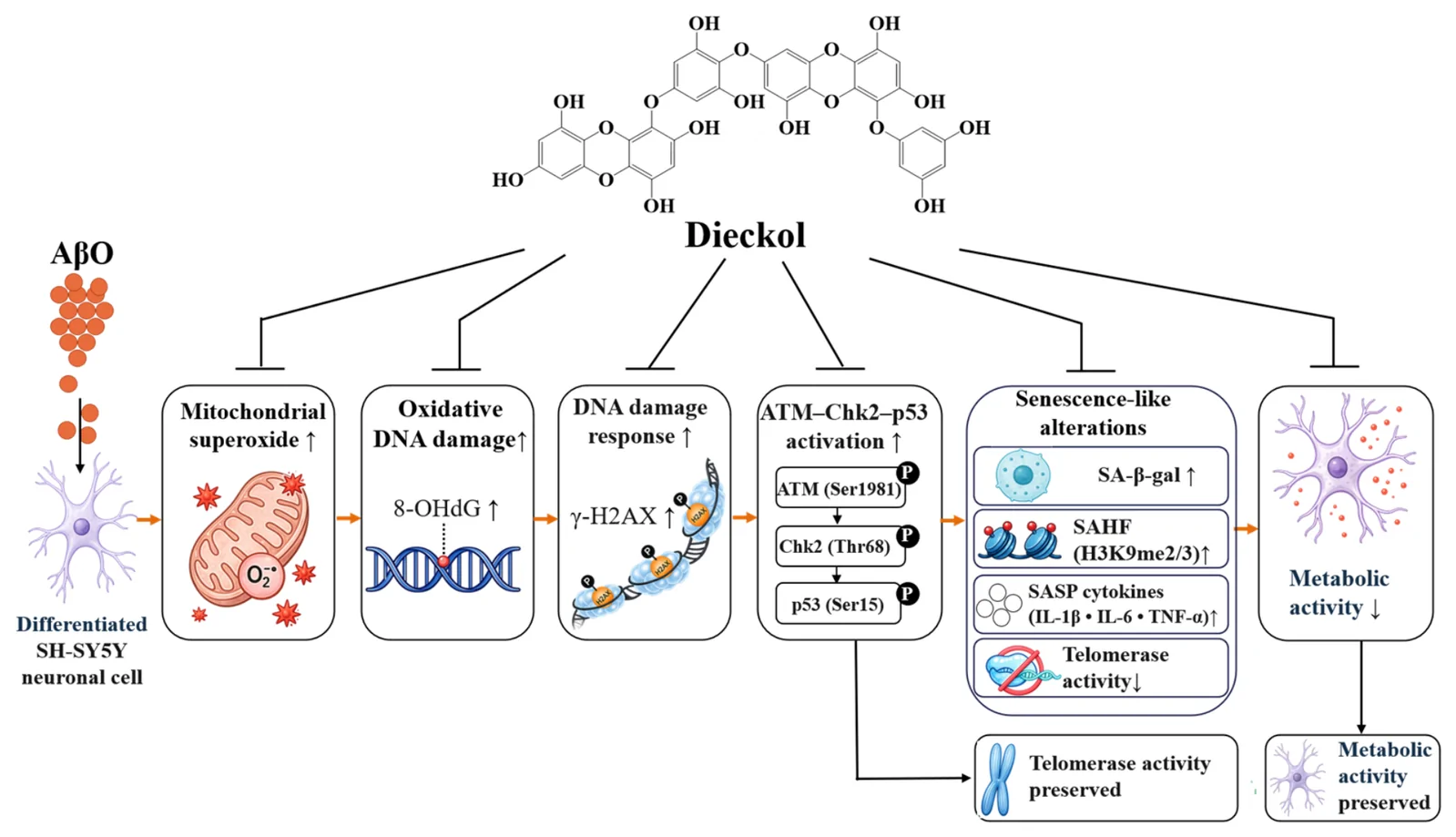
Proposed interpretative model of the protective effects of dieckol against AβO-induced injury in SH-SY5Y cells. Dieckol and its chemical structure are shown at the top of the schematic. AβO exposure increases mitochondrial superoxide accumulation and oxidative DNA modification, as indicated by elevated 8-OHdG levels. These alterations are accompanied by increased γ-H2AX-associated fluorescence and activation of ATM–Chk2–p53 signaling. Persistent oxidative and genotoxic stress may further promote a senescence-like stress response characterized by increased SA-β-gal activity, H3K9me2/3-associated chromatin remodeling, enhanced secretion of SASP-related cytokines, and reduced telomerase activity, ultimately contributing to impaired cell viability. Dieckol attenuates these interconnected oxidative, DNA damage response-associated, and senescence-associated alterations, partially restores telomerase activity, and preserves cell viability during AβO exposure. Arrows indicate proposed stimulatory or activating effects, whereas blunt-ended lines indicate proposed inhibitory effects. The connecting arrows represent hypothesized relationships based on the observed parallel changes and existing literature and do not indicate causal links directly demonstrated in the present study.

In conclusion, the present findings demonstrate that dieckol protects differentiated SH-SY5Y cells against AβO-induced cytotoxicity and concurrently attenuates mitochondrial superoxide accumulation, oxidative DNA damage, DNA damage response signaling, and multiple senescence-like alterations. These coordinated effects are compatible with a possible relationship among mitochondrial oxidative stress, DNA damage response signaling, and senescence-associated stress; however, they do not establish a causal or sequential mechanism. Figure 7 therefore summarizes an interpretative model that should be tested in future studies using temporal and pathway-specific interventions.

## 4. Materials and Methods

### 4.1. Culturing of SH-SY5Y Cells and Induction of Neuronal Differentiation

Human SH-SY5Y neuroblastoma cells (CRL-2266; ATCC, Manassas, VA, USA) were cultured in DMEM/F12 medium (Cat. No. SLM-243-B; Sigma-Aldrich, St. Louis, MO, USA) supplemented with 10% fetal bovine serum (Cat. No. F2379; Sigma-Aldrich), 1% nonessential amino acids (Cat. No. 11140050; Thermo Fisher Scientific, Waltham, MA, USA), 100 U/mL penicillin, and 100 μg/mL streptomycin (Cat. No. 15140148; Thermo Fisher Scientific). Cells were maintained at 37 °C in a humidified atmosphere containing 5% CO_2_. For neuronal differentiation, SH-SY5Y cells were seeded in 100-mm culture dishes at a density of 1 × 10^6^ cells/dish and maintained in complete growth medium. When the cultures reached approximately 40–50% confluence, the medium was replaced with complete growth medium containing 10 μmol/L all-trans retinoic acid (RA; Cat. No. R2625; Sigma-Aldrich). Cells were continuously exposed to RA for five days, with fresh RA-containing medium provided every 48 h. The RA-based differentiation procedure was adapted from a previously established approach [31]. Cultures were examined by phase-contrast microscopy during the differentiation period. The appearance and elongation of neurite-like processes extending from the cell soma were used solely as qualitative indicators of a neuron-like phenotype. Neurite length was not quantitatively measured, and cellular morphology was not analyzed as an experimental endpoint in the present study. After five days of RA exposure, the differentiated cells were gently detached using 0.05% trypsin in phosphate-buffered saline (PBS; pH 7.4) and reseeded in six-well plates at a density of 1 × 10^5^ cells/well. Cells were allowed to reattach for 24 h before subsequent experimental treatments were initiated.

### 4.2. Aβ_42_ Oligomer Preparation

Aβ_42_ oligomers (AβOs) were prepared according to the previously described protocol [32]. Lyophilized synthetic Aβ_42_ peptide (Cat. No. ab120301; Abcam, Cambridge, UK) was allowed to equilibrate at room temperature for 30 min before the vial was opened to minimize moisture condensation. The peptide was dissolved in ice-cold 1,1,1,3,3,3-hexafluoro-2-propanol (HFIP; Cat. No. 105228; Sigma-Aldrich) to a concentration of 1 mmol/L and incubated at room temperature for 30 min to disrupt pre-existing peptide aggregates. The resulting solution was divided into single-use aliquots in low-protein-binding polypropylene tubes. HFIP was allowed to evaporate at room temperature, and residual solvent was subsequently removed under vacuum until a thin, transparent peptide film was obtained. The dried peptide films were sealed and stored at −20 °C until use. Immediately before oligomer preparation, each peptide film was reconstituted in dimethyl sulfoxide (DMSO; Cat. No. 2650; Sigma-Aldrich) to obtain a 5 mmol/L Aβ_42_ stock solution. The solution was vortexed and sonicated in a water bath for 10 min to ensure complete peptide dispersion. Ice-cold phenol red-free Ham’s F-12 medium (Cat. No. PM150813; Procell Life Science & Technology Co., Ltd., Wuhan, China) was then added to achieve a final AβO concentration of 100 μmol/L. After gentle mixing, the preparation was incubated without agitation at 4 °C for 24 h to promote oligomer formation. The resulting AβO preparation was freshly diluted in culture medium to the required treatment concentration and used immediately. To minimize preparation-to-preparation variability, all peptide films were generated from an HFIP-treated AβO stock, divided into single-use aliquots, and subjected to identical reconstitution, dilution, incubation, and handling conditions. The resulting preparations were used immediately without refreezing. Direct biochemical or biophysical characterization of oligomer formation and size distribution was not performed in the present study.

### 4.3. Establishment of AβO-Induced Cellular Injury and Treatment Protocols

Dieckol (Cat. No. HY-147059; MedChemExpress LLC, Monmouth Junction, NJ, USA) and azeliragon (Cat. No. HY-50682; MedChemExpress LLC) were dissolved in DMSO to prepare stock solutions of 50 and 100 mmol/L, respectively. The solutions were thoroughly mixed by vortexing and briefly sonicated when necessary. Stock solutions were protected from light, divided into single-use aliquots, and stored at −20 °C. Immediately before each experiment, the stocks were diluted in culture medium to the required concentrations and visually inspected for precipitation. The final DMSO concentration was adjusted to 0.5% (*v*/*v*) in all experimental and corresponding vehicle-control groups [33]. This concentration was not independently evaluated against a DMSO-free condition across all cellular endpoints; therefore, subtle vehicle-related effects cannot be completely excluded. Nevertheless, the use of an identical DMSO concentration in all groups allowed treatment effects to be assessed relative to a common vehicle background.

To establish the cellular injury model, differentiated SH-SY5Y cells were exposed to 20 μmol/L AβO for 24 h, based on previously reported protocols for Aβ-mediated neurotoxicity [34]. This condition was selected following preliminary viability experiments because it produced a reproducible but incomplete reduction in MTT activity, thereby retaining a sufficient number of viable cells for evaluating pharmacological protection. For intervention experiments, differentiated cells were pretreated with dieckol (10, 30, or 50 μmol/L) or azeliragon (100 μmol/L) for 24 h. AβO was subsequently added at a final concentration of 20 μmol/L, and the cells were incubated for an additional 24 h at 37 °C in the continued presence of the corresponding compound. The dieckol concentration range was selected based on previous studies demonstrating cellular tolerance at concentrations up to 50 μmol/L in N2a and SweAPP-overexpressing N2a cells and protective activity against AGE–RAGE-associated injury [13,14]. Azeliragon was included at 100 μmol/L based on previous AD-related cellular studies reporting attenuation of NLRP3-associated signaling, apoptosis-related responses, and excessive ROS production [35]. Parallel cultures treated with dieckol or azeliragon in the absence of AβO were included to assess compound-associated cellular metabolic activity.

### 4.4. Assessment of Cell Viability

Cell viability was indirectly assessed on the basis of cellular metabolic activity using the 3-(4,5-dimethylthiazol-2-yl)-2,5-diphenyltetrazolium bromide (MTT) assay (Cat. No. M2003; Sigma-Aldrich), with minor modifications to a previously described method [36]. Differentiated SH-SY5Y cells were plated in 96-well plates at a density of 5 × 10^3^ cells/well and permitted to adhere overnight. To examine the individual effects of AβO and the test compounds, cells were treated for 24 h with AβO (20 μmol/L), dieckol (10, 30, or 50 μmol/L), or azeliragon (100 μmol/L). For evaluation of cytoprotection, cells were preincubated with dieckol or azeliragon for 24 h and subsequently challenged with 20 μmol/L AβO for a further 24 h while the corresponding compound remained present. At the end of the treatment period, MTT prepared in minimum essential medium was added to each well at a final concentration of 0.5 mg/mL Following incubation at 37 °C for 4 h, the MTT-containing medium was removed, and the resulting formazan crystals were solubilized in 200 μL of DMSO. Absorbance was recorded at 570 nm using a SpectraMax M5 multimode microplate reader (Molecular Devices, Sunnyvale, CA, USA). Each biological experiment was performed in technical triplicate, and the mean absorbance of the technical replicates was used as the value for that experiment. Cellular metabolic activity was calculated relative to the corresponding vehicle-treated control group, which was assigned a value of 100%.

### 4.5. Measurement of Mitochondrial Superoxide

Mitochondrial superoxide accumulation was evaluated using the MitoSOX™ Red mitochondrial superoxide indicator (Cat. No. M36008; Invitrogen, Carlsbad, CA, USA) [37]. Following the indicated treatments, cells were rinsed with phosphate-buffered saline (PBS; pH 7.4) and incubated with 5 μmol/L MitoSOX Red for 10 min at 37 °C in the dark. After two washes with PBS, nuclei were counterstained with 5 μg/mL Hoechst 33342 (Cat. No. H3570; Invitrogen) for 10 min at room temperature in the dark. Fluorescence images were acquired using a Leica fluorescence microscope (Leica Microsystems, Wetzlar, Germany) under identical acquisition settings for all treatment groups. MitoSOX and Hoechst signals were detected in the red and blue channels, respectively. Hoechst staining was used to enumerate nuclei for normalization of the MitoSOX fluorescence signal and was not used as a quantitative assessment of nuclear morphology or apoptosis. The integrated MitoSOX fluorescence intensity was quantified using ImageJ software (version 1.53; National Institutes of Health, Bethesda, MD, USA) in five randomly selected fields per biological experiment. For each field, the integrated MitoSOX fluorescence intensity was divided by the number of Hoechst-positive nuclei. The normalized values from the five fields were then averaged to generate one value for each biological replicate. Results were normalized to the vehicle-treated control group and expressed as fold change.

### 4.6. Assessment of Senescence-Associated β-Galactosidase Activity

Senescence-associated β-galactosidase (SA-β-gal) activity was examined as a marker of senescence-like alterations using a commercial staining kit (Cat. No. 602010; Cayman Chemical, Ann Arbor, MI, USA), in accordance with the manufacturer’s protocol and a previously described method [38]. Following the indicated treatments, cells grown in six-well plates were rinsed twice with PBS and fixed with 1 mL of 1× fixative solution for 15 min at room temperature. The fixative was then removed, and the cells were washed twice with PBS before the addition of 1 mL of freshly prepared SA-β-gal staining solution. Plates were covered to limit evaporation and incubated at 37 °C for 16 h in a CO_2_-free incubator. Cells displaying blue cytoplasmic staining were classified as SA-β-gal-positive and visualized using an Olympus CKX53 light microscope (Olympus Corporation, Tokyo, Japan). For each biological experiment, SA-β-gal-positive cells and total cells were counted in five randomly selected fields. The percentage of positive cells was calculated for each field, and the mean percentage across the five fields was used as one biological replicate for statistical analysis.

### 4.7. Quantification of Senescence-Associated Secretory Phenotype (SASP)-Associated Cytokines

Following the indicated treatments, conditioned culture media were harvested for quantification of the SASP-associated pro-inflammatory cytokines interleukin-1β (IL-1β), interleukin-6 (IL-6), and tumor necrosis factor-α (TNF-α). Cytokine levels were measured using human IL-1β (Cat. No. ab214025), IL-6 (Cat. No. ab178013), and TNF-α (Cat. No. ab181421) ELISA kits (Abcam) in accordance with the manufacturer’s protocols. Optical density was recorded at 450 nm using a SpectraMax M5 multimode microplate reader (Molecular Devices), and cytokine concentrations were interpolated from the respective standard curves. After collection of the conditioned media, the corresponding cells were lysed, and total cellular protein was quantified using a BCA protein assay kit (Cat. No. ab102536; Abcam) according to the manufacturer’s instructions. The amount of each cytokine recovered from each well was normalized to the total cellular protein content of the corresponding cell lysate and expressed as pg/mg protein. Each sample was assayed in technical triplicate, and the mean of the technical replicates was used as the value for each biological experiment.

### 4.8. Determination of Relative Telomerase Activity

Relative telomerase activity was determined using the TeloTAGGG™ Telomerase PCR ELISA kit (Cat. No. 11854666910; Roche Diagnostics GmbH, Mannheim, Germany) according to the manufacturer’s instructions. Following the indicated treatments, cells were counted, and 2 × 10^5^ cells from each sample were collected, washed with ice-cold phosphate-buffered saline, and resuspended in 200 μL of chilled lysis reagent. Cell suspensions were maintained on ice for 30 min and subsequently centrifuged at 16,000× *g* for 20 min at 4 °C. The resulting supernatants were collected and immediately subjected to the telomeric repeat amplification protocol (TRAP). For each reaction, an identical volume of cell extract (3 μL) was combined with 25 μL of the supplied reaction mixture, and the final volume was adjusted to 50 μL with sterile water. Thus, assay input was standardized according to cell number, lysis volume, and extract volume; no additional normalization to total protein content was performed. Telomerase-mediated extension and PCR amplification were performed according to the kit protocol. The amplified products were subsequently denatured and hybridized with a digoxigenin-labeled telomere-specific probe. Hybridized products were captured on streptavidin-coated microplates through the biotin-labeled primer and detected using a peroxidase-conjugated anti-digoxigenin antibody and tetramethylbenzidine substrate. Absorbance was measured at 450 nm with a reference wavelength of 690 nm using a SpectraMax M5 multimode microplate reader (Molecular Devices). Heat-inactivated cell extracts were included as negative controls, whereas the cell extract supplied with the kit served as the positive control. Each biological sample was analyzed in technical triplicate, and the mean absorbance of the technical replicates was used as the value for that biological experiment. After subtraction of the corresponding background absorbance, telomerase activity was normalized to the vehicle-treated control group, which was assigned a value of 100%.

### 4.9. Immunofluorescence Staining

Immunofluorescence staining was performed to evaluate di-/tri-methylated histone H3 lysine 9 (H3K9me2/3), Ser139-phosphorylated histone H2AX (γ-H2AX), and 8-hydroxy-2′-deoxyguanosine (8-OHdG). Following treatment, cells were fixed with 4% paraformaldehyde for 15 min and permeabilized and blocked for 1 h in PBS containing 0.3% Triton X-100 and 5% normal goat serum (Cat. No. G9023; Sigma-Aldrich). Cells were incubated overnight at 4 °C with anti-H3K9me2/3 (clone 6F12; Cat. No. 5327; Cell Signaling Technology, Danvers, MA, USA; 1:100), anti-phospho-H2AX (Ser139; Cat. No. 2577; Cell Signaling Technology; 1:800), or anti-8-OHdG (clone E-8; Cat. No. sc-393871; Santa Cruz Biotechnology, Dallas, TX, USA; 1:100). After washing with PBS, cells were incubated for 1 h at room temperature in the dark with the corresponding secondary antibody: Alexa Fluor 488-conjugated anti-mouse IgG (Cat. No. 4408; Cell Signaling Technology; 1:1000), Alexa Fluor 594-conjugated anti-rabbit IgG (Cat. No. 8889; Cell Signaling Technology; 1:1000), or Alexa Fluor 488-conjugated goat anti-mouse IgM (Cat. No. A21042; Invitrogen; 1:500), respectively. Cells were then washed with PBS and counterstained with DAPI (Cat. No. 4083; Cell Signaling Technology). Images were acquired using an Olympus FV3000 confocal laser-scanning microscope (Olympus Corporation, Tokyo, Japan), with identical acquisition settings maintained across all treatment groups for each marker. Five randomly selected fields from each of five independent biological experiments were analyzed using ImageJ software (version 1.53; National Institutes of Health, Bethesda, MD, USA). For H3K9me2/3 and 8-OHdG, the background-subtracted integrated fluorescence intensity across each field was normalized to the number of DAPI-positive nuclei. For γ-H2AX, DAPI images were used to define nuclear regions of interest, and fluorescence quantification was restricted to these regions. Following background subtraction, the integrated fluorescence intensities within the nuclear regions were summed for each field and divided by the number of DAPI-positive nuclei in that field. The resulting values from the five fields were averaged to generate one value for each biological replicate. Results were normalized to the vehicle-treated control group and expressed as fold change.

### 4.10. Assessment of DNA Damage Response Signaling

Activation of ATM–Chk2–p53 signaling was evaluated using phospho/total protein ELISA assays. After the indicated treatments, cells were washed with ice-cold PBS and lysed in the kit-provided lysis buffer containing protease and phosphatase inhibitors. Lysates were gently agitated at 2–8 °C for 30 min and clarified by centrifugation at approximately 16,000× *g* for 10 min at 4 °C. The supernatants were collected and maintained on ice until analysis. Phosphorylated and total protein levels were measured using Phospho-ATM (Ser1981)/Total ATM (Cat. No. ab279740), Phospho-Chk2 (Thr68)/Total Chk2 (Cat. No. ab279762), and Phospho-p53 (Ser15)/Total p53 (Cat. No. ab279878) ELISA kits (Abcam), according to the manufacturer’s instructions. Phosphorylated and total forms of each protein were measured in their respective wells using the same lysate preparation and dilution. Following color development with tetramethylbenzidine substrate, the reactions were terminated with stop solution, and absorbance was measured immediately at 450 nm using a SpectraMax M5 microplate reader (Molecular Devices). After background subtraction, phospho-ATM/total ATM, phospho-Chk2/total Chk2, and phospho-p53/total p53 ratios were calculated from the corresponding absorbance values. Each biological experiment was analyzed in technical triplicate, and the mean of the technical replicates was used as one biological replicate. Data from five independent biological experiments were normalized to the vehicle-treated control group and expressed as fold change.

### 4.11. Statistical Analysis

Data are expressed as the mean ± standard deviation (SD) from five independent biological experiments (*n* = 5). Technical replicates or field-level measurements were averaged within each experiment to generate a single value for each biological replicate; only these biological replicate values were included in the statistical analyses. Differences among treatment groups were evaluated using one-way analysis of variance (ANOVA), followed by Tukey’s multiple-comparisons test. Statistical analyses were performed using SigmaPlot version 14.0 (Systat Software, Inc., San Jose, CA, USA). A two-sided *p* value < 0.05 was considered statistically significant.

## Data Availability

The original contributions presented in this study are included in the article. Further inquiries can be directed to the corresponding authors.
